# Forensic psychiatric care professionals’ experiences of communicating with foreign patients and their relatives: the importance of language and culture

**DOI:** 10.3389/fpsyt.2025.1643806

**Published:** 2025-08-07

**Authors:** Omed Abdul Rahman, Eirini Alexiou, Alessio Degl’Innocenti, Christopher Holmberg, Patricia Olaya-Contreras

**Affiliations:** ^1^ Institute of Health and Care Sciences, Sahlgrenska Academy, University of Gothenburg, Gothenburg, Sweden; ^2^ Department of Forensic Psychiatry, Sahlgrenska University Hospital, Gothenburg, Sweden; ^3^ Centre for Ethics, Law and Mental Health, Department of Psychiatry and Neurochemistry, Institute of Neuroscience and Physiology, Sahlgrenska Academy, University of Gothenburg, Gothenburg, Sweden; ^4^ Västra Götaland Region, Regionhälsan, Gothenburg, Sweden; ^5^ Department of Psychotic Disorders, Sahlgrenska University Hospital, Gothenburg, Sweden

**Keywords:** communication, cultural competence, forensic psychiatric care, immigrant, language barrier, qualitative content analysis

## Abstract

**Background:**

Clear and effective communication between patients and professionals in forensic psychiatric care is central to providing accurate information about care and treatment and building trust. Limited language proficiency hinders mutual understanding, making therapeutic interactions significantly more complex. In Sweden, there has been an increase in the number of non-Swedish-speaking immigrants during the last decade, which further emphasizes this important aspect. The aim of this study was to explore the experiences of forensic psychiatric professionals and identify factors influencing effective communication in their interactions with patients and their relatives who have limited proficiency in Swedish.

**Methods:**

Nine professionals specializing in forensic psychiatry— including physicians, registered nurses, and counselors—participated in deep individual interviews. Qualitative content analysis was conducted inductively, following the Standards for Reporting Qualitative Research.

**Results:**

One overarching theme emerged—effective communication is the foundation of care and treatment but difficult to separate from culture and translation. Four categories were identified (1): importance of satisfactory communication for effective patient care, (2) challenges and paradoxes of interpreter use, (3) cultural understanding as a guide for deep communication in forensic psychiatry relationships, and (4) health literacy needs of patients and relatives to comprehend treatment.

**Conclusion:**

To improve forensic psychiatric care, healthcare providers must adopt structured language assistance measures, such as interpreter services and cultural competency training for professionals. Participants emphasized the importance of integrating cultural awareness in care delivery and ensuring precise communication of complex psychiatric concepts to minimize misunderstandings and enhance patient engagement. Family education and health literacy are crucial in fostering comprehension and optimizing interpreter use, enabling consistent and clear communication. Implementing these measures can lead to a more inclusive healthcare environment, ensuring equal access to mental health services for linguistically diverse patients.

## 1 Introduction

In Sweden, forensic psychiatric care is mandatory for individuals who have committed a crime and meet specific legal criteria, including having a severe psychiatric disorder (SFS, 1991:1129). To protect society, special court supervision was introduced in Sweden in 1992, enabling courts to monitor individuals sentenced to forensic psychiatric care, particularly in cases where there is a risk of relapse into serious criminal behavior (1). Additionally, In Sweden’s multicultural society, forensic psychiatric care faces unique challenges stemming from diverse cultural backgrounds (2), making communication essential. Health care professionals use communication to assess and evaluate patients’ mental state, provide care, and interact with families. The overarching goal of Swedish health policy and health care services is to ensure equitable care for the entire population, including for patients in forensic psychiatric care (3). However, inequalities in health care between foreign-born and Swedish-born individuals persist (4). The growing number of patients with foreign backgrounds in forensic psychiatric care necessitates additional language support and culturally adapted care. This need is underscored by the increase in the proportion of foreign-born persons in Sweden, which rose from 11% in 2000 to 20% in 2021, representing an increase of approximately 2 million people (5). The rise in patients with foreign backgrounds may introduce cultural and language barriers that diminish the effectiveness of therapy and prolong the duration of treatment (6). The median duration of forensic psychiatric treatment is 58 months, significantly longer than in general psychiatric care (7). The length of treatment depends on mental health, treatment needs, and legal factors. There is no fixed time frame, and courts review the need for continued care biannually (7).

Various barriers to communication can affect an individual’s ability to describe their health status and share information about themselves. A lack of knowledge about such barriers can also affect an individual’s ability to receive customized information regarding care planning and the legal aspects of forensic psychiatric care. A lack of awareness about the barriers also impedes healthcare professionals from initiating tailored, personalized conversations with patients and their relatives (8, 9). Research has indicated that patients who are not proficient in the dominant language used in a health care environment feel less safe on such wards than patients of non-foreign background, possibly due to a lack of understanding about care- and treatment-related information (6). The most common type of psychiatric disorder in forensic psychiatric care in Sweden is psychotic disorders, with schizophrenia being the most common among both men and women (10). These disorders are associated with cognitive impairments that may affect patients’ understanding of care-related communication (11). Research has additionally shown that culturally adapted psychosocial interventions can effectively support patients with schizophrenia, especially considering cognitive and contextual barriers to engagement (12). In that vein, culture encompasses values, language, traditions, and behaviors and thus shapes how individuals perceive and make sense of their experiences, particularly in mental health care. Cultural and social norms, meanwhile, influence meaning-making, which affects how illness and treatment are experienced.

Intercultural communication involves exchanging information between individuals from different cultural backgrounds, thereby making cultural competence essential for health care professionals to provide quality care (13). Effective intercultural communication also helps to bridge differences and fosters mutual understanding. Nevertheless, a recent review has revealed only limited research on the communication-related requirements of patients of foreign background and health care professionals, as well as the strategies employed to address language-related and cultural challenges encountered in accessing and delivering psychiatric care (9). Language and culture are deeply interconnected, for language not only facilitates communication but also transmits cultural norms and traditions. Cultural competence involves knowledge and skills that enable health care professionals to interact effectively with individuals from different cultural backgrounds (14). In forensic psychiatry, understanding and respecting cultural differences are fundamental for providing patient-centered care. Equitable care in a multicultural society requires professionals to reflect on perceptions of patients regarding factors such as age, gender, and social background. Cultural sensitivity, closely linked to cultural competence, involves interacting with awareness and respect for cultural differences (15).

Forensic psychiatric care, as a form of coercive care, is influenced by cultural factors that shape both its implementation and how it is perceived by patients and their families. This influence underscores the need for professionals to prioritize verbal and nonverbal communication to better understand patients’ cultural backgrounds and care needs. Meanwhile, patients’ awareness of their rights and obligations, as well as information about their care and confidentiality, is pivotal for patients of foreign backgrounds. Forensic psychiatric professionals need to be aware of these factors to prevent misunderstandings and ensure that patients’ needs for high-quality care are met.

In our study, we aimed to explore the experiences of forensic psychiatric care professionals in communicating with patients, as well as with their relatives, from foreign backgrounds and with limited proficiency in Swedish.

## 2 Methods

### 2.1 Study design

We conducted an exploratory descriptive qualitative study that followed an inductive approach and used semistructured interviews for data collection. Inductive qualitative analysis involves examining the information provided without reference to any specific theoretical framework and without necessarily testing a theory (16, 17). Such an inductive approach enabled us to explore and identify the experiences of forensic psychiatric professionals in communicating with patients and their relatives of foreign background and with limited proficiency in Swedish.

### 2.2 Recruitment

To recruit participants for the study, we employed a purposive sampling technique (18). None of us, the authors, had any prior relationships with any participants in the study. Recruitment was conducted by specific research representatives that caters for all forensic psychiatric care units and who are responsible for disseminating information about ongoing research and for recruiting participants. Posters with information about the study were also placed in the units, which allowed health care professionals to receive written information about the study. That step enabled them to directly contact the author responsible for conducting the interviews to request more information and express their interest in participating.

The inclusion criteria for participation included at least one year of work experience in forensic psychiatric care, direct involvement in caring for and treating patients with limited proficiency in Swedish in forensic psychiatry, and experience with interacting and communicating with patients and their relatives. In total, nine health care professionals were recruited: two counselors, two psychiatrists, a psychologist, and four registered nurses, some with specialist training in psychiatric care. All participants had extensive experience in both general psychiatric care and forensic psychiatric care, with overall work experience ranging from 1–30 years. All participants maintained close contact with patients receiving forensic psychiatric care in both inpatient and outpatient settings (see **Table 1**). The inclusion of participants with substantial experience in forensic psychiatric care strengthens the credibility of the study by ensuring that the data is grounded in informed, context-specific knowledge. Their familiarity with institutional procedures and exposure to a range of clinical situations enhances the dependability and confirmability of the findings, as their accounts are more likely to reflect consistent and verifiable patterns. Moreover, the depth and relevance of their insights contribute to the overall validity of the study by supporting the generation of trustworthy and transferable knowledge.

**Table 1:** Participants’ characteristics (*N* = 9).

Participant	Occupation	Gender	Years working in forensic psychiatric care
1	Counselor	Woman	7
2	Registered nurse	Woman	3
3	Registered nurse	Woman	1.5
4	Counselor	Woman	6
5	Consultant psychiatrist	Woman	2
6	Registered nurse	Woman	1
7	Consultant specialist in psychiatry	Man	3
8	Registered nurse with specialist training in psychiatric care	Woman	10
9	Psychologist	Man	12

### 2.3 Data collection

The participants were interviewed individually between June and August 2024. All interviews were conducted by OAR, a male registered mental health nurse with an MSc and several years of experience in Swedish forensic psychiatric care, as well as formal training in how to conduct interviews. During data collection he worked clinically in the clinic, while concurrently pursuing a PhD. Prior to recruitment no author had a therapeutic relationship with prospective participants. OAR introduced himself as a clinician-researcher and emphasized the voluntary nature of participation to minimize perceived coercion. To refine the interview guide and ensure that questions elicited open, non-leading responses aligned with the study’s aims, a pilot interview was conducted with one of the authors (CH). The interviewer and two other authors (CH and POC) reviewed the questions together to confirm their relevance and clarity. Throughout the research process, the interviewer was supported by team members.

A semistructured interview guide was developed that reflected different themes in its questions, including “How do you perceive the role of communication in your professional practice?” and “Do you use any tools or aids to communicate with patients who have not sufficiently mastered the Swedish language (e.g., interpreter, written information, or websites)?” (see **Supplementary File 1**). The interviews were conducted in a private conference room to ensure a setting free from disturbances. Each session lasted approximately 40 to 60 minutes. The interview questions were designed to elicit detailed accounts of the participants’ experiences and interactions with patients in forensic psychiatric settings who possess limited proficiency in the Swedish language. Participants were encouraged to engage in open-ended dialogue, allowing them to elaborate on their experiences of communication with both patients and their relatives under these linguistic constraints. During the transcription process, conducted in parallel with the interviews, the authors reviewed the full content to verify quality and ensure trustworthiness. After conducting nine interviews, the team discussed and determined that data saturation had been reached when no new information related to the research aim emerged (19). This indicated that additional interviews would not provide further insights, and thus, no additional participants were interviewed. Each interview was recorded using a high-quality audio-recording device (dictaphone) and transcribed with SONIX software. Prior to the recording of the interviews, the participants were asked to provide information regarding their years of experience, occupation, and gender. To ensure the protection of research subjects’ data and maintain the confidentiality of their information, a unique code was assigned to each interview. During the analysis, the participants’ responses were referenced using the coding system. After the transcription process, a cross-checking of the audio files with the original text was conducted to ensure accuracy and clarity.

### 2.4 Research team and reflexivity

The research team comprised individuals with diverse professional and personal backgrounds, contributing to the reflexive rigor of the study. Although no personal relationships existed between the authors and participants, the interviewer had prior professional interactions with some participants. To mitigate potential interpretive bias and enhance *confirmability*, the team engaged in open discussions during data analysis to ensure that participants’ perspectives were accurately represented. Notably, four of the authors were not born in Sweden but had long-standing experience in Swedish psychiatric care, and one had prior research experience with non-Swedish populations. This diversity in professional and cultural backgrounds enriched the analytical process, supporting *credibility* and *transferability* by enabling a multifaceted interpretation of the data. The team remained reflexively aware of the sensitivity of the topic and committed to presenting participants’ experiences authentically and respectfully.

### 2.5 Data analysis

In an inductive content analysis of the participants’ responses, the transcribed interviews were repeatedly reviewed to ensure completeness and data fidelity. All authors on the research team participated in this process. The research team comprised the following authors: OAR (Master of Science in Mental Health Nursing, doctoral student), EA (Ph.D., forensic psychiatrist), ADI (Ph.D., clinical psychologist), CH (Ph.D., registered nurse), and POC (Ph.D., registered nurse). All the authors, except for the doctoral student, have published qualitative studies, and contributed different disciplinary perspectives to interpreting the data. The interviewer (OAR) initially coded the data and assigned preliminary codes, with additional researchers (EA, AA, CH, and POC) independently conducting the data analysis. The analysis was conducted as follows: First, the meaning units—i.e., sentences or short paragraphs related to the study’s aim—that is, the health care professionals’ experiences with forensic psychiatric patients of foreign backgrounds and their relatives—were identified in the interview transcripts. Then, these meaning units were condensed into shorter meaning units and thereafter coded. Those codes were subsequently categorized, which yielded an overarching theme (20). Multiple reviews by the authors ensured accuracy and credibility. Discussions were held at several stages to achieve consensus, ensured data saturation, and mitigate researcher bias. An iterative process involving regular comparisons of data for patterns and differences strengthened the confirmability of the findings. Engaging multiple researchers with various professional backgrounds in the coding process enhances trustworthiness by incorporating varied perspectives, enriching interpretation, and supporting credibility. Cross-verification of data among researchers strengthens dependability and confirmability, ensuring consistency and mitigating interpretive bias. Distributing the analytical workload enabled more extensive engagement with the data, contributing to the study´s overall validity.

### 2.6 Ethical considerations

All participants received oral and written information about the study at the time of the interviews and were asked to participate in the study by signing consent forms. They were also informed that they could withdraw from the study at any time without specifying a reason. The privacy of participants was also considered when handling personal data, and no participants’ personal data were reported at the individual level. The Standards for Reporting Qualitative Research (21) were followed to enhance transparency by providing explicit criteria for reporting important components of the qualitative study (see **Supplementary File 2**). The study was approved by the Swedish Ethical Review Authority (No. 2023–04821–01).

## 3 Results

The analysis of the information given by participants revealed an overarching theme and four categories that elucidated how language barriers impact health care professionals’ experiences with communicating with forensic psychiatric patients of foreign background and their relatives. Those categories, together with their respective subcategories, encapsulate the core findings of the analysis and are presented in **Table 2**. The overarching theme, “Effective communication is a foundation of care and treatment but difficult to separate from culture and translation,” reflects how communication is both central to care and complicated by linguistic and cultural dimensions. It serves as a unifying perspective across the four categories.

**Table 2:** Theme, categories, and subcategories that emerged from qualitative inductive content analysis.

Theme: Effective communication is a foundation of care and treatment but difficult to separate from culture and translation.	
Categories	Subcategories
1. The importance of effective communication for quality patient care	• Communication as a crucial tool for psychiatric forensic care • Language barriers as impeding the building of relationships with patients and their relatives • Risk of misinterpreting patients’ conditions due to difficulties with communicating • The need for written and adapted communication with patients and their relatives
2. The paradox of using interpreters	• Challenges and opportunities with on-site interpretation • Challenges and opportunities with remote interpretation • Strategies to ensure safe, accurate interpretation
3. Cultural understanding as a guide for deep communication in therapeutic relationships	• How health care professionals create an understanding of patients’ cultural backgrounds • Family-focused culture and cultural distrust of health care
4. Health literacy needs of patients and families to comprehend treatment	• Limited health literacy among patients and their relatives • The need for knowledge and support to build an understanding of treatments among patients and their relatives

As for the four categories within the overarching theme, the first category highlights the critical role of effective communication in ensuring quality care by emphasizing how communication-related challenges can hinder the relationship between health care professionals and patients, which may lead to misdiagnosis and inadequate treatment. Next, the second category explores various situations in which interpreters are used and delineates both the opportunities and obstacles that arise when they facilitate communication between health care professionals and patients. Meanwhile, the third category addresses the significance of cultural competence as a foundation for deeper, more meaningful communication within health care relationships. Last, the fourth category addresses the need for health literacy among patients and their families, which was viewed as being essential for understanding the treatment process. The categories and their subcategories are described in detail in the following subsections.

### 3.1 The importance of effective communication for quality patient care

Effective communication is paramount for ensuring quality care for patients. The first category highlights how communication-related challenges can significantly impact the relationship between health care professionals and patients. On the one hand, poor communication may lead to misunderstandings, misdiagnosis, and inappropriate treatment plans. On the other, ensuring clear, accurate, empathetic communication is essential for building trust, understanding patients’ needs, and delivering effective care. The participants especially emphasized the importance of communication as a crucial tool in caring for and treating patients with psychiatric diagnoses. They also highlighted that language barriers hinder the establishment of relationships with patients and their relatives and noted that communication plays a vital role in facilitating change and fostering mutual understanding between health care professionals and patients, which ultimately improves the quality of care. Effective communication is not just about exchanging information; it’s about creating a shared understanding and working together toward the best possible outcome for the patient. This insight highlights the transformative power of communication in achieving collaborative and patient-centered care.

#### 3.1.1 Communication as a crucial tool for psychiatric forensic care

The participants stressed the significant role of effective communication encompassing both verbal and nonverbal modalities as a fundamental tool for providing forensic psychiatric care. The described approach ultimately aims to enhance the targeted treatment and overall well-being of patients throughout the therapeutic process. Several participants noted the importance of effective communication between health professionals and patients to reduce misunderstandings and promote patient safety:

(Communication) is our tool, you could say. However, I think it’s both what you say and what you don’t say. So, it’s communication in a very broad sense and the whole impression that’s more than just verbal aspects, I think. [ … ]. But it’s absolutely crucial in all our work. (Participant 5)

The participants suggested that health care professionals have to understand patients’ symptoms and behaviors in order to provide appropriate care. Therefore, patients whose communication is impaired require more time to understand communicated messages, process them, and respond in keeping with the additional work demands. Conversations should allow sufficient time to ensure that the information is repeated and that patients receive the opportunity to reflect at their own pace. They also highlighted the need for established communication with both patients and their relatives as a crucial aspect of the treatment process:

Communication is the main source of information for assessing patient health. Information from health professionals and conversations with patients and relatives are crucial, so communication is very important. (Participant 7)

This reflection underscores the multifaceted nature of communication in psychiatric forensic care. It is not merely about exchanging information but also about establishing a therapeutic alliance that fosters trust and understanding.

#### 3.1.2 Language barriers impeding the building of relationships with patients and their relatives

Health care professionals identified language barriers among relatives as a significant obstacle to establishing genuine relationships and effectively communicating with patients of foreign background. Those barriers impede the fostering of mutual understanding, hinder opportunities for meaningful interactions, and obstruct effective communication between patients and their social environment, including their relatives. The barriers also restrict the creation of an open channel of communication between health care professionals and their relatives:

It’s like this: If there are initial language difficulties, then you never come into contact with them [the relatives]. How is a relationship supposed to be formed? (Participant 1)

Working with language to change other people is central. I work a lot with communication to find common ground, to create opportunities, and to make life possible for both patients and other people. Our [health care professionals’] role is to make ourselves understandable to others by understanding our patients based on the knowledge that we have and helping others to understand. (Participant 9)

The participants considered proficiency in their own language to be fundamental, along with awareness of each patient’s linguistic and cultural background, for those factors influence verbal expression and communication dynamics. They added that health care professionals should confirm their understanding of patients’ responses and explain the steps that they take to address barriers to communication to give patients the opportunity to express their needs and be understood accordingly:

You need to have good knowledge of your own language first of all. You also need to know what the patient’s language is and what their cultural origin is and things that I see as having a connection with it, such as how verbal you are and how communication can be conveyed. (Participant 8).

Language barriers affect not only direct communication between healthcare professionals and patients but also profoundly impact relationships with patients’ relatives. This can lead to misunderstandings and a sense of isolation for both patients and their families. To overcome these obstacles, healthcare professionals attempted to establish a relationship with relatives from the initial contact. However, language barriers impeded this process creating a significant obstacle. Therefore, health care professionals must be linguistically and culturally competent. By actively working to understand and adapt to patients’ linguistic and cultural backgrounds, healthcare professionals can create a more inclusive and supportive care environment.

#### 3.1.3 Risk of misinterpreting patients’ conditions due to difficulties with communicating

The participants cautioned that language difficulties increase the risk of misinterpreting and even misdiagnosing patients’ conditions. They highlighted that difficulties with communication frequently present a significant risk of misinterpreting patients’ health status, which can hinder accurate diagnosis and effective treatment:

It’s important to master the language. It’s part of the treatment process. I have to explain clearly what treatment the patient is receiving and communicate continuously so as not to create misunderstanding. The treatment will fail if we misunderstand each other. (Participant 3)

Based on their experiences, the participants emphasized that language barriers and difficulties in self-expression can cause frustration and irritability among patients. Although such reactions may be misinterpreted by health care professionals as signs of mental health deterioration, it is the language barriers that are the underlying problem:

Patients who express frustration, irritation, and irritability are likely to be assessed as deteriorating or as being agitated or anxious. It may be that they have difficulties with communication and hence frustration, which we then interpret as deterioration. (Participant 1)

Some participants expressed concerns regarding the potential misinterpretation of patients’ health status due to language barriers. In particular, individuals who do not speak Swedish or who have limited proficiency in the language may be perceived as being more ill than they actually are. That linguistic challenge can hinder patients’ ability to articulate their needs and desires effectively, which can impact the gravity with which their expressions are received:

There’s a risk that people who don’t speak Swedish or speak it poorly or inadequately may be perceived as being sicker than they are and may find it more difficult to express their wishes and be taken seriously. (Participant 5)

These insights underscore the critical need for healthcare professionals to develop robust communication strategies that account for linguistic diversity. By doing so, they can mitigate the risks of misinterpretation and ensure that all patients receive accurate diagnoses and appropriate care.

#### 3.1.4 The need for written and adapted communication with patients and their relatives

The participants expressed concerns about whether patients with psychiatric diagnoses have adequate access to knowledge and information about their diagnoses and treatment. The fear is particularly pronounced among patients who do not fully understand the Swedish language. As a consequence, patients of foreign background are at a disadvantage and often receive suboptimal treatment and care due to language barriers and cultural differences:

I don’t feel that forensic psychiatry patients receive sufficient information about their treatments or diagnoses, but it’s worse for people whose primary language isn’t Swedish, who receive less information that’s adapted. (Participant 9)

Several participants who revealed that patients often lack sufficient information regarding their diagnoses and treatments emphasized the importance of providing written materials in the patient’s native language in order to ensure comprehension. Written information should also be provided whenever there is any doubt about a patient’s understanding:

The information that we provide to our patients in Swedish should definitely be available in several languages. Yes, but it isn’t. (Participant 4)

Several participants underscored the importance of providing written information and adapting multilingual resources as essential tools to enhance understanding and communication between patients and health care professionals. Moreover, digital tools such as Google Translate, among others, were mentioned by participants as potentially useful tools for enhancing communication:

A patient may say yes, that he has understood and so on, but you might still doubt whether it’s truly been conveyed correctly. But then I think that it [communication] still works with Google Translate [ … ]. I think it’s also a lot about the information really being correct. (Participant 2)

Ensuring that patients fully understand their diagnoses and treatments is necessary for effective care. Providing written information in multiple languages and utilizing digital translation tools can significantly enhance communication and understanding. This approach not only empowers patients but also helps bridge the gap caused by language barriers, leading to better health outcomes.

### 3.2 The paradox of using interpreters

Despite a clear need for interpretation when language barriers exist, participants questioned the effectiveness of using interpreters. These interpreters were professional, trained, and certified by the health care system. The participants reported mixed experiences with interpreters; some preferred having an interpreter present in person, whereas others found remote interpretation to be an effective means of facilitating and understanding patients’ communication. Moreover, participants who noted that the logistical challenges of relying on in-person interpreters (e.g., hindering the interpretation process) were more likely to understand the need for remote interpreters.

#### 3.2.1 Challenges and opportunities with on-site interpretation

Several participants underscored the importance of having an on-site interpreter versus a remote telephone interpreter. They highlighted that an on-site interpreter facilitates direct communication, which allows accurate contextual understanding, timely corrections, and appropriate interventions. However, the participants also reported mixed experiences with using interpreters. Although the majority preferred on-site interpreters due to their ability to minimize misunderstandings and reduce the need for repetition, some acknowledged both challenges and opportunities associated with in-person interpretation:

Conversations are not as easy when you use an interpreter. You don’t get concrete information especially not with a telephone interpreter. I’d rather have an interpreter in the room. (Participant 4)

The on-site interpreter makes the conversation much easier. There will be much less mishearing and much less interruption in the conversation. (Participant 9).

The preference for on-site interpreters highlights the importance of direct, face-to-face communication in healthcare settings. This approach not only enhances the accuracy of information exchange but also fosters a more personal and trusting relationship between patients and healthcare professionals.

Participants also identified logistical challenges with using interpreters, including the difficulty of securing a quiet, suitable location in the hospital for conversations involving the interpreter, patients, and health care professionals. The booking system was reported to present considerable difficulties as well. The participants noted that, owing to those logistical challenges, on-site interpreters were booked less frequently:

It’s logistically difficult to find an opportunity to meet in another room and to coordinate it with maybe four more people and book it in a new system. [ … ]. However, I think that both for me and for others it’s probably been an obstacle sometimes, unfortunately. (Participant 9)

#### 3.2.2 Challenges and opportunities with remote interpretation

According to the participants, the use of telephone interpreters increased following the COVID-19 pandemic. When interpreters work over the telephone, participants identified patients’ personal conditions and technological obstacles as primary challenges to maintaining an uninterrupted conversation. They described telephone interpretation as problematic also due to poor reception, technical difficulties, the inability to observe facial expressions and maintain eye contact, and the risk of mishearing. Together, those factors worsen communication between interpreters and health care professionals, which impacts the comprehension of patients’ situations. Consequently, telephone interpreters often fail to convey accurate information to both patients and health care professionals. The participants reported both the advantages and disadvantages of that approach:

I use telephone interpreters. The patient is usually here and the interpreter elsewhere. I think it’s more complicated; they don’t hear very well or don’t see facial expressions or gestures. Sometimes they don’t hear (the interpreter); the reception is bad, they hack, or the patient interrupts. (Participant 6)

The increased reliance on telephone interpreters during the pandemic has highlighted significant challenges in maintaining effective communication. These challenges underscore the need for improved technological solutions and training to ensure that remote interpretation can meet the standards of in-person interpretation.

Remote interpretation was widely viewed as challenging due to the risk of misinterpretation or omission. The majority viewed remote interpretation as more of a hindrance than a resource. Even so, some patients were more likely to be vocal when telephone interpreters were used instead of on-site interpreters:

It’s better to have an interpreter on-site. Since the pandemic, there’s been more interpretation by phone; it can work well, but it’s better to have an interpreter on-site. I find that some patients become more vocal with telephone interpreters. (Participant 5)

Using telephone interpreters also presented the need for repeating back information, which was reported to generally enhance comprehension for both patients and health care professionals. However, despite the increased use of remote interpretation following the pandemic, advanced technology to that end has not been implemented, and telephone interpretation remains the sole alternative:

I find that when you get information over the phone, the interpreter is forced to repeat it. (Participant 9).

When you get information over the phone, the interpreter is forced to ask you to repeat the information. You could solve that problem with better technology around it all. Video calls haven’t been tested. (Participant 9)

#### 3.2.3 Strategies to ensure safe, accurate interpretation

The participants also stated that they typically prepare interpreters before assignments and emphasize the interpreter’s role as a neutral intermediary. Patients are also informed about the interpreter’s role. Participants highlighted the importance of interpreters who are sensitive to cultural aspects and can accurately convey culturally specific expressions used by patients, which strengthens the accuracy of translated information:

I’ve always thought that I should prepare the interpreter so that they know their mission and are prepared for assignments in which they will be neutral mediators in some way, which can be a reasonable approach. If the patient also understands what the interpreter’s task is, then it might be helpful. (Participant 9)

Sometimes, despite the interpreter translating a common language, it appears that the same language can have different interpretations, leading to misunderstandings for the patient within the context. Nevertheless, participants also noted that some experienced interpreters know the forensic psychiatric system and the specific patient’s culture when providing interpretive services, which ensures an appropriate interpretation of the feelings and reactions of patients from specific cultural backgrounds:

Some interpreters are incredibly skilled and very good at handling the rather special role of being interpreters and can also help in a good way to translate cultural aspects. (Participant 5)

The preparation of interpreters and their sensitivity to cultural nuances are crucial for ensuring accurate and effective communication in healthcare settings. By understanding the cultural context and specific needs of patients, interpreters can bridge the gap between healthcare professionals and patients, leading to better patient outcomes and a more inclusive healthcare environment.

### 3.3 Cultural understanding as a guide for deep communication in therapeutic relationships

Understanding the cultural background of patients was considered to be important for health professionals who work closely with patients. Cultural beliefs can pose barriers to communication, more so than purely linguistic language difficulties. A thorough understanding of cultural differences and nuances can significantly enhance the quality of communication between health care professionals and their patients. The participants implied that cultural awareness was essential for building meaningful, trustworthy relationships in their specialized field, which could lead to better patient outcomes and more accurate assessments.

#### 3.3.1 How health care professionals create an understanding of patients’ cultural backgrounds

The participants emphasized the importance of understanding the cultural backgrounds of patients in order to better comprehend the goals and wishes of patients and their relatives. One participant highlighted the need to imagine oneself in the patient’s cultural context in order to foster a deeper understanding of their situation. Another participant noted that cultural beliefs among patient of foreign background can pose a greater barrier to communication than language difficulties alone:

It’s not only language that’s needed; it’s also about understanding a cultural context that you need to familiarize yourself with. When I worked with assessments, I was careful to read up on patients´ cultural backgrounds often. (Participant 4)

It’s very important for us [health care professionals] to understand the patient’s cultural background and how it works in the specific family to get an understanding of their situation. Which direction should we work toward? What are the patient’s goals? Are they compatible with what the family wants? (Participant 2)

If you talk about language and culture, I think that cultural beliefs are a greater obstacle to communication than perhaps the linguistic difficulties that are purely linguistic. (Participant 2)

#### 3.3.2 Family-focused culture and cultural distrust of health care

The participants also underscored the distrust of health care systems among some patients and their families. Cultural distrust in health care describes a lack of trust in the health care system and/or in medical professionals, often stemming from historical and/or personal experiences of discrimination, mistreatment, and/or misunderstanding. Such distrust highlights the need for health care professionals to (re)build trust and explain the health care process clearly to both patients and their relatives, who often play a central role in decision-making, including about health care-related decisions:

In purely cultural terms, many people live much closer to their families, so the family doesn’t take as much help from social services because you’re in the family. It’s perhaps more difficult for many people who aren’t from Sweden to understand that you have the opportunity to just call the facility and say that you want to meet us, that you might not do it [seek treatment] if you’re not invited. (Participant 5).

Building trust with patients and their families is essential for adequate person-centered care and effective healthcare delivery. Understanding and addressing cultural distrust can help healthcare professionals create a more welcoming and supportive environment.

One participant noted that some family members refrain from seeking help or establishing contact with health care providers. Moreover, some patients experience shame associated with receiving forensic psychiatric care and prefer to shield their relatives from the burden of engaging with forensic psychiatric staff. As a result, those patients often navigate the health care system independently and may avoid seeking support from social services:

There are many patients who are ashamed of being in forensic psychiatry and who want to spare their relatives the burden of having to deal with us [forensic psychiatric staff]. (Participant 2)

Moreover, patients and their relatives of foreign background were reported to often be unfamiliar with forensic psychiatry. The patients are generally young at the time of admission and lack prior contact with that specific part of Sweden’s health care system. At the same time, many patients have had experience with legal authorities, social services, and correctional institutes, which can lead to preconceptions about forensic psychiatric care, including that it is associated with authoritarianism. Those perceptions can result in mistrust or apprehension toward forensic health care services. The participants described frequently needing to explain the entire system to patients because they are generally unaccustomed to that approach:

I also have to work with an interpreter when I have conversations with them [patients]. It’s the same thing there [the explanation of the forensic care], because I also have to explain the whole system, and people may think that some [patients] have had a different relationship with the authorities or health care or whatever it may be and aren’t at all used to using interpreters. (Participant 4)

Healthcare professionals must be proactive in educating patients and their families about the forensic psychiatric system. Clear communication and cultural sensitivity can help dispel misconceptions and build trust, ultimately leading to better patient outcomes.

### 3.4 Health literacy needs of patients and families to comprehend treatment

The fourth and final category emphasizes the need for health literacy among both patients and their families to comprehend treatment. It elucidates the participants’ identification of needs to enhance their understanding of every patient’s disease and prognosis. Several participants noted that although contact with patients and their family members is limited, both patients and their relatives greatly need education. Moreover, the understanding and interpretation of the oral and written information provided about their mental disorders was described as being important for patients and their family members to understand the severity of their disorders and to accept treatment.

#### 3.4.1 Limited health literacy among patients and their relatives

The participants emphasized that both patients and their families need health literacy in order to comprehend their diagnoses and treatments. They advocated educating patients and their families after the diagnosis has been communicated and understood in order to ensure proper support for patients. Knowledge and information about the disorder are essential to create understanding among patients and their family members:

Knowledge about the disorder and an understanding of why you [the health care professional] do what you do with the patient are important for relatives, too. (Participant 5)

The participants noted that patients and their relatives did not know where to seek information related to the patient’s condition or how to understand what such information means in practical terms. They advised patient education and family education as pivotal strategies for comprehending what to do after diagnosis, including by creating forums for patients and families to share their experiences and connect with one another. Such initiatives could foster mutual understanding and improve the effectiveness of written communication, which could ultimately enhance care outcomes:

There are many patients who haven’t received information or haven’t been able to absorb the information that they have received from us or simply don’t know how to seek information. (Participant 2)

Improving health literacy among patients and their families is essential for achieving the best possible results in patient health and well-being. Educating them about diagnoses and treatments can empower them to make informed decisions, follow treatment plans accurately, and engage more effectively with healthcare providers for better support.

#### 3.4.2 The need for knowledge and support to build an understanding of treatment among patients and their relatives

The participants highlighted the lack of a structured forum where family members could connect with other families and patients could engage with fellow patients. Moreover, the participants recognized the need for a well-organized educational program for both patients and their relatives to support them after diagnosis:

After you’ve been diagnosed, you should attend patient and relatives training, which was very organized, though I think we could be better at it. Since then, relatives have been together with other relatives and patients. Was there someone who held the conversation as well? I think it’s very good, because many people need to meet others who are in the same situation. (Participant 5)

Participants additionally described experiences from other psychiatric departments in which health care professionals prioritize strategies to support relatives. That approach enables family members to identify early warning signs and promptly seek help when the patient’s mental state begins to deteriorate:

It is important to prioritize contacts with relatives. In other types of care for psychosis, it’s crucial to support relatives, teach them how to handle things, and educate them in early signs so that relatives are alerted in time. But here [forensic psychiatry], there’s an ambition now to get better; we don’t have training for relatives and leave it to interest groups. (Participant 7)

## 4 Discussion

The purpose of our study was to explore the experiences of forensic psychiatric care professionals with communicating with patients and their relatives of foreign background and who have limited proficiency in the Swedish language. The findings highlight the critical role of suitable communication—that is, communication that is understandable in its content and format in relation to the specific situation (e.g., that communication differs in an hour-long session with a psychologist vs. communication with a nurse during medication administration vs. communication during a meeting with a psychiatrist about one’s diagnosis). The participants also emphasized that for communication to be suitable and appropriate in facilitating care and treatment, they needed to consider and adapt to the fact that patients had severe psychiatric disorders and language limitations. Many participants emphasized that the limited language proficiency of patients and their relatives posed significant challenges to communication and resulted in an impaired development of a therapeutic relationship. Some even noted that language barriers could lead patients to be misjudged as more mentally impaired than they were. It has been confirmed that language barriers in forensic psychiatric care result in poor communication between health care professionals and patients, which negatively impacts the satisfaction of both parties and can subsequently impair the quality of care, jeopardize patient safety, and lead to errors in treatment and assessment (22, 23). From a therapeutic perspective, verbal and written communication is crucial for assessing diagnoses, treating patients and evaluating treatment outcomes. This study focuses on oral and written communication, particularly how these forms of communication affect the interactions during treatment with patient and their relatives. However, non-verbal cues like body language and facial expressions are also significant, providing insights into emotional states and intentions. Future research should incorporate both verbal and non-verbal communication for a comprehensive understanding of communication dynamics in forensic psychiatry.

Regarding the use of interpreters, participants reported mixed experiences. Most favored on-site professional interpreters over remote options due to advantages such as minimizing misunderstandings and the need for repetition. However, one participant reported that patients sometimes opened up more during phone-interpreted sessions. Those findings are reinforced by the results of a review examining the use of interpreters in various health care settings in relation to their effects on the quality of care from the perspectives of both health care professionals and patients (24). Similar to our results, the review indicated that in-person professional interpreters yielded the highest satisfaction levels and most effective communication outcomes among patients. Using interpreters in clinical consultation and clinical work is a strategy to facilitate communication by patients of foreign background and their relatives. More recently, other research has emphasized important aspects of using interpreters, including that interpreters should be booked on the basis of the patient’s primary language, not nationality, and that health professionals should use professional and knowledgeable interpreters who can provide adequate communication for patients with limited language skills (25). Many patients of foreign background who are treated in forensic psychiatric care may be of the same nationality but speak different languages and dialects. Therefore, when booking interpreters for patients, the patient’s first language and dialect are considered to be important to ensure that the patient receives an interpreter with the right language skills.

Added to linguistic challenges, participants identified cultural beliefs as a more significant barrier to communication. They stressed the importance of understanding patients’ cultural backgrounds, particularly their roles in their families and the patients’ own perceptions of their mental disorders. Some noted that families might rely solely on their own networks and avoid seeking external health care support, which further complicates their engagement in the patients’ care. In agreement with our results, previous research has emphasized the need for cultural competence training for health care professionals and the introduction of cultural competence in psychiatric health care to address those differences and build bridges with patients of different cultural backgrounds and their families (26–29).

A family-focused culture emphasizes the importance of family relationships and responsibilities. In such cultures, family members often play a central role in decision-making, including about health care decisions. As a result, patients may rely heavily on their family’s opinions and support when it comes to medical treatment and care (30).

A cultural distrust of health care describes a lack of trust in the health care system and/or medical professionals, often due to historical and/or personal experiences of discrimination, mistreatment, and/or misunderstanding. Such distrust can lead to reluctance in seeking medical help, in following medical advice, and/or in fully disclosing health information to health care providers. When those two factors are combined, they can create significant barriers to effective health care communication and treatment. Patients from family-focused cultures who also have a cultural distrust of health care may be less likely to engage with health care providers, follow medical advice, and/or seek necessary treatments. Previous research has emphasized the importance of transparent health care organizations in fostering a culture of person-centered practice. Transparency enables patients to actively participate in their care and treatment by providing them with accurate information about their diagnoses and available treatment options. In turn, the approach promotes accountability and helps to cultivate trust in the health care system (31, 32).

### 4.1 Strategies for overcoming communication barriers

Several researchers have proposed evidence-based strategies and actionable frameworks for health care stakeholders to enhance the clarity, accessibility, and effectiveness of communication to develop patient-centered care. For instance, the FRAME framework includes five strategies: familiarizing oneself with the patient’s communication skills and resources, reducing the speed of speech, assisting with message construction, using a mixed communication approach, and engaging the patient. Those strategies are recommended for health care professionals seeking to communicate effectively with patients experiencing communication difficulties and to avoid barriers to the patient’s understanding and safety in ways that impact health outcomes (29, 32, 33). Those studies using this type of framework have highlighted that familiarizing oneself with patient communication can serve as a bridge between health care professionals and patients. Therefore, health care professionals should assess patients’ communication skills, including their use of assistive devices (e.g., hearing aids and glasses) and their ability to express themselves. Moreover, they should contribute to improving the health literacy of patients as well as their relatives (29, 32). Prior to a planned conversation, professionals should evaluate patients’ communication strategies and adapt accordingly in consideration of any and all barriers. Those findings are consistent with what previous research has shown (9).

Furthermore, our results emphasize the importance of the cultural competence of health care professionals and of mutual understanding between the interpreter and the health care professional and between the interpreter and the patient (26, 27, 29). Previous research has shown that patients as well as health and social care practitioners tend to prefer mental health services that reflect both the client’s language and cultural background. Although professional interpreters often become necessary, their use is not always favored and can present its own set of challenges (9).

In line with our results, past research has highlighted the importance of collaboration between interpreting agencies and health care professionals to ensure and provide high-quality care and that those improvements are crucial to providing good care to patients of foreign background who face language barriers (22, 23, 31). Professional interpreters, who play an important role in the interpretation process by arriving on time, checking in with patients, and determining with patients whether the language and dialect are correct as a means to enhance successful interactions (34), need to be trained in medical terminology and cultural diversity. Moreover, our study showed that clarifying the roles of all parties is important for establishing an appropriate relationship and for ensuring safe, accurate interpretations during consultation, as is consistent with previous findings (34, 35).

## 5 Strengths and limitations

A major strength of our study was its focus on a diverse group of health care professionals, which allowed a comprehensive understanding of the communication-related challenges faced in forensic psychiatric care. This breadth of experience contributes to a more comprehensive understanding of the research topic. However, the participants are predominantly female and from a single region, which limits the inclusion of male perspectives and those of healthcare providers from other regions. The use of in-depth interviews enabled the collection of rich, detailed data that highlight the critical role of effective communication and the impact of cultural and linguistic barriers on patient care.

Our results also have several limitations. For one, the single-hospital setting may limit the transferability of our findings. As a result, findings might mostly be transferable within countries with similar justice and health care systems. Our study focused on a single forensic psychiatric clinic, which may also limit the applicability of the findings to other health care environments. Beyond that, our findings only reflect the participants’ perceptions and personal experiences and attitudes. Although data saturation was reached after nine interviews, the small sample size remains a limitation, potentially affecting the variability of insights. Future research could benefit from more diverse samples and from including multiple sites to increase the transferability of the results.

## 6 Conclusion

In sum, the findings underscore effective communication’s critical role in providing care to patients with psychiatric disorders and language limitations in order to prevent health inequalities. Significant challenges posed by limited language proficiency can hinder therapeutic relationships and lead to misjudgments about patients’ mental impairments. Using interpreters, although generally beneficial at times, brought about mixed experiences, with participants’ preferring on-site interpreters in order to minimize misunderstanding. This study highlights the need for a “structured interpreter system,” which involves selecting competent and certified interpreters. Further, these interpreters should meet with the therapist prior to meeting the patient, to understand the diagnosis, treatment and plans. This preparation ensures the accurate delivery of information from the clinician and staff to the patient and their relatives. Cultural beliefs were also identified as substantial obstacles to communication. On that topic, understanding patients’ cultural backgrounds and their perceptions of mental illness is crucial for effectively engaging patients, as well as their relatives, in their care and support. Our results additionally emphasize that health care professionals need cultural competence training to bridge gaps in their relationships with patients from diverse cultural backgrounds. This training should involve patient representatives and relatives discussing various cultural knowledge and beliefs, aiming to understand reactions and plan appropriate actions. Significant awareness of patient´s oral and written language barriers is required, as these barriers can hinder accurate diagnosis and assessment. Particularly, written tests may be misinterpreted due to patients´ lack of language proficiency, leading to misleading assessments and treatment evaluations. Additionally, utilizing alternative technological tools, both written and oral, may verify therapeutic communication. Last, fostering transparency within health care organizations can enhance patients’ participation and build trust, which ultimately improves the quality and accessibility of forensic psychiatric care for patients with foreign backgrounds who have limited skills in the Swedish language.

## Data Availability

The raw data supporting the conclusions of this article will be made available by the authors, without undue reservation.

## Supplementary material

Supplementary File 1 Semistructured Interview Guide.

Supplementary File 2 Standards for Reporting Qualitative Research Checklist.
