# Supplementary material for: Forensic psychiatric care professionals’ experiences of communicating with foreign patients and their relatives: the importance of language and culture
Source: Front Psychiatry. 2025 Aug 7;16:1643806. doi: 10.3389/fpsyt.2025.1643806 (PMC12368502; doi:10.3389/fpsyt.2025.1643806)
Supplement: Supplementary File 1 — Semistructured Interview Guide. [file Supplementaryfile1.docx]

# Supplementary Files

# Supplementary File 1: Semistructured Interview Guide

***Introduction and information to participants***

Thank you for participating in our study.

Your perceptions and experiences are valuable information for us to better understand forensic psychiatric care. The following questions focus on your experiences with and perceptions of communication with patients and their relatives who have a foreign background and whose native language is not Swedish.

You have the right to refuse to answer any question without giving a reason. You also have the right to end the interview at any point without explanation.

Do you have any questions about what I have said so far? Is it okay for you if we begin now?

***Descriptive Information***

- What is your occupation?

(e.g., doctor, physiotherapist, psychologist, registered nurse, other profession)

- For how many years have you worked in forensic psychiatric care?

Number of years: ______ years

- Do you have specialist training in your profession?

No, or, if yes, specify:______________

I will begin audio-recording the interview now.

*[Start audio recording]*

***General Communication and Information in Forensic Psychiatric Care***

1. How do you perceive the role of communication in your professional practice?
2. How do you perceive the role of language and language skills in your professional practice?
3. Do you use any tools or aids (e.g., interpreter, written information, or websites) to communicate with patients who have not sufficiently mastered the Swedish language?
4. Are there any communication aids (e.g., written information in other languages) that you lack?
5. Is there anything that you lack for communicating or providing information to patients?
6. How do you perceive that patients’ proficiency in Swedish impacts their care and communication? Please provide examples.
7. How do you perceive that the proficiency of patients’ relatives in Swedish impacts patients’ care? Please provide examples.
8. Do you feel that patients in forensic psychiatric care receive sufficient information about their treatment (e.g., about their diagnosis and type of care)?
9. In your experience, what consequences have resulted from inadequate communication with patients or relatives? Can you provide an example?

***Interactions and Attitudes***

1. How do you perceive patient–health care professional interactions in forensic psychiatry for patients whose first language is not Swedish?
2. How do you perceive patients’ opportunities to practice their religion or celebrate specific holidays with their relatives?

***Caring and Therapeutic Conversations***

1. How do you ensure that patients whose first language is not Swedish understand your message or information during a conversation? Can you provide an example?
2. What do you think is required to establish a caring relationship and therapeutic conversation with patients whose first language is not Swedish?
3. From your professional perspective, how do you view communication and conversations?

# Supplementary File 2: Standards for Reporting Qualitative Research Checklist

| **Item** | **Description** | **Location in manuscript** |
| --- | --- | --- |
| 1. Title | Identifies the study as qualitative and describes the topic | Title Page |
| 2. Abstract | Summarizes background, purpose, methods, findings, and conclusions | Abstract |
| 3. Problem formulation | Rationale, significance, prior work described | Introduction |
| 4. Purpose or research question | Clearly stated aim of study | End of Introduction |
| 5. Qualitative approach and research paradigm | Qualitative content analysis mentioned; no explicit paradigm | Methods – Study design |
| 6. Characteristics and reflexivity of researcher(s) | Multidisciplinary team described; partial mention of reflexivity; could be more explicit | Methods – Data analysis |
| 7. Context | Context of forensic psychiatry in Sweden described | Introduction |
| 8. Sampling strategy | Inclusion criteria, purposive sampling, and saturation discussed | Methods – Recruitment and participants |
| 9. Ethical issues | Ethics approval, informed consent, and privacy addressed | Methods – Ethical considerations |
| 10. Data collection methods | In-depth interviews and iterative transcription process described | Methods – Data collection |
| 11. Data collection instruments and technology | Interview guide mentioned but not detailed; SONIX software used | Methods – Data collection |
| 12. Units of study | Detailed table of participants’ roles, genders, and years of experience | Methods – Participants |
| 13. Data processing | Transcription, coding, anonymization, and data verification described | Methods – Data analysis |
| 14. Data analysis | Inductive process, multiple coders, and iterative checks explained | Methods – Data analysis |
| 15. Techniques to enhance trustworthiness | Multiple analysts, consensus discussions, and iterative analysis noted | Methods – Data analysis |
| 16. Synthesis and interpretation | Clear presentation of overarching theme and categories | Results – Overview and category headings |
| 17. Links to empirical data | Extensive use of direct quotations from participants | Results – All subsections |
| 18. Integration with prior work and discussion of transferability | Discussed in context with FRAME and interpreter literature; limitations acknowledged | Discussion |
| 19. Limitations | Clearly addressed in a dedicated section | Discussion – Limitations |
| 20. Conflicts of interest | Clearly addressed in a dedicated section | Statements section – Prior to the references |
| 21. Funding | Clearly addressed in a dedicated section | Statements section – Prior to the references |
